# Correlation of X-ray diffraction signatures of breast tissue and their histopathological classification

**DOI:** 10.1038/s41598-017-13399-9

**Published:** 2017-10-11

**Authors:** Robert M. Moss, Amany S. Amin, Chiaki Crews, Colin A. Purdie, Lee B. Jordan, Francesco Iacoviello, Andrew Evans, Robert D. Speller, Sarah J. Vinnicombe

**Affiliations:** 1 Department of Medical Physics and Biomedical Engineering, University College London, Gower Street, London, WC1E 6BT UK; 2 Royal London Hospital, Barts Health NHS Trust, Whitechapel Road, London, E1 1BB UK; 3 NHS Tayside Pathology Department, Ninewells Hospital and Medical School, Dundee, DD1 9SY UK; 4 Department of Chemical Engineering, University College London, Gower Street, London, WC1E 6BT UK; 5 Division of Cancer Research, University of Dundee, Ninewells Hospital and Medical School, Dundee, DD1 9SY UK

## Abstract

This pilot study examines the correlation of X-ray diffraction (XRD) measurements with the histopathological analysis of breast tissue. Eight breast cancer samples were investigated. Each sample contained a mixture of normal and cancerous tissues. In total, 522 separate XRD measurements were made at different locations across the samples (8 in total). The resulting XRD spectra were subjected to principal component analysis (PCA) in order to determine if there were any distinguishing features that could be used to identify different tissue components. 99.0% of the variation between the spectra were described by the first two principal components (PC). Comparing the location of points in PC space with the classification determined by histopathology indicated correlation between the shape/magnitude of the XRD spectra and the tissue type. These results are encouraging and suggest that XRD could be used for the intraoperative or postoperative classification of bulk tissue samples.

## Introduction

Breast cancer is the most common form of cancer in the UK with around 55,000 cases diagnosed each year^1^. Research has highlighted the importance of tumour-associated stroma in breast cancer initiation and progression^2^. A major component of such stroma is abnormal collagen, the amount and orientation of which can directly influence epithelial growth and transition as well as response to neoadjuvant therapy^3,4^. However, conventional X-ray imaging is unable to interrogate the nature of stromal collagen since there is little or no contrast between the X-ray attenuation properties of normal and abnormal collagen. Development of a technique that could identify abnormal collagen could have a profound impact on breast cancer diagnosis and treatment.

Early breast cancer treatment usually involves breast conserving surgery (BCS) where the cancerous tissue is removed from the patient. Histopathological analysis is carried out on the excised tissue postoperatively. The tissue is prepared for microscopic analysis starting with dissection of the fresh sample, chemical fixation to prevent decay, further dissection and sampling of regions of interest, processing of the tissue into paraffin wax and production of stained microscope slides. The purpose of the analysis is to classify the tumour and to ensure that all of the malignancy has been removed. Where cancerous tissue is found at the margins of the resected tissue, the patient may be required to undergo further surgery.

Conventional histopathological analysis relies on examining a thin (4 µm thick) slice of tissue based on its micro-structure (cellular scale) through the use of optical microscopy. However, changes in the nano-scale structure associated with disease^5^ can also be used to discriminate between different tissue types, suggesting that X-ray scattering techniques, such as Small Angle X-ray Scattering (SAXS)^6–8^ and X-ray diffraction (XRD)^9–11^ are suitable for this purpose.

XRD is a well-established technique which is used to analyse the atomic or molecular structure of materials. When X-rays interact with matter they can be scattered by the electrons in the constituent atoms. If the resultant (or scattered) X-ray has the same phase and energy as the initial X-ray then it is said to have scattered coherently. Diffraction occurs when X-rays are scattered coherently from different atomic or molecular layers within a material separated by a distance (*d*). The results is constructive interfere at certain angles (2*θ*), where Bragg’s Law (Equation (1)) is satisfied, giving rise to intense cones of radiation.

1 $$\lambda \,=\,2d\,\sin \,\theta $$

**Figure 1:**
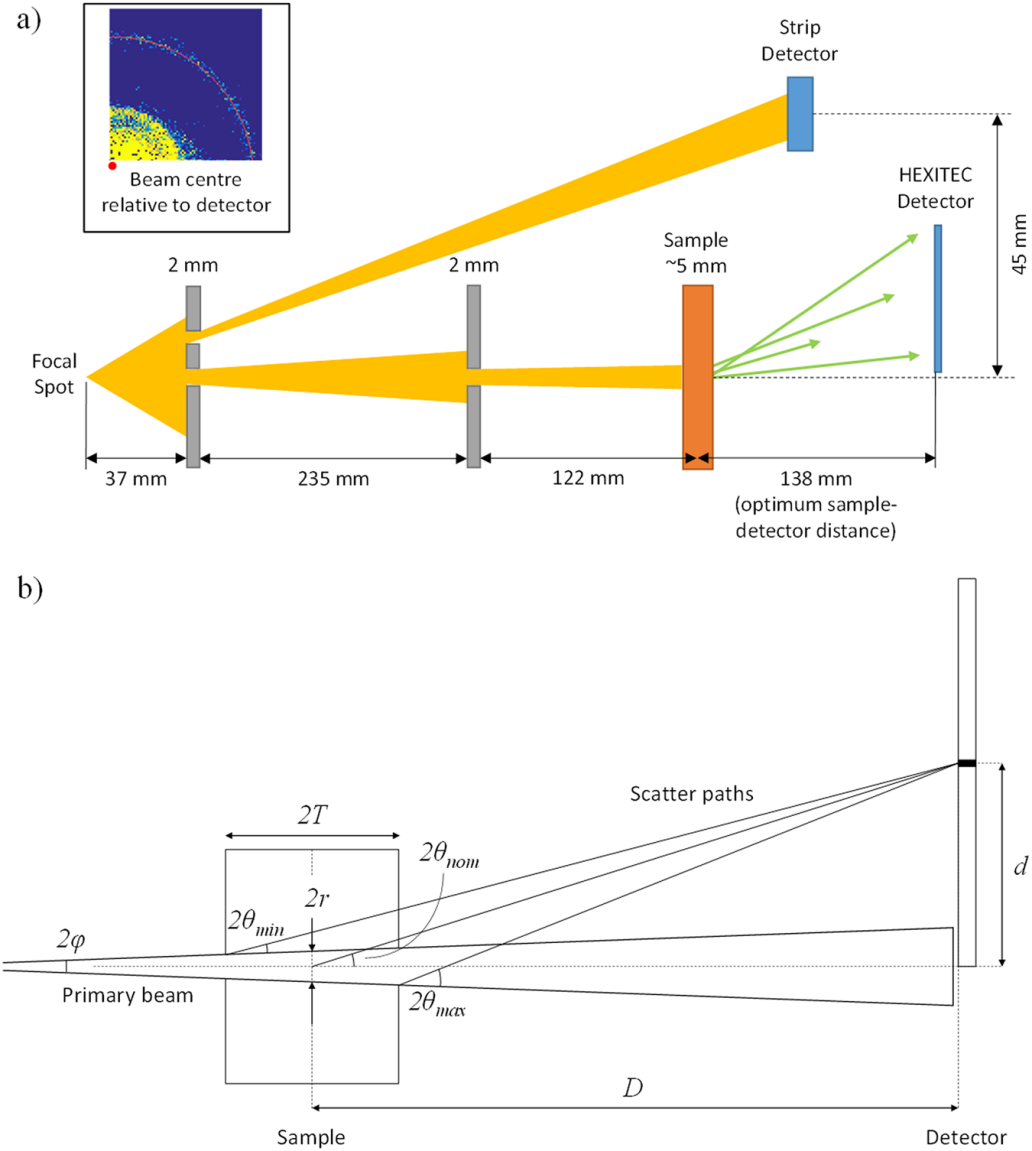
(**a**) Schematic representation of the *miniPixD* instrument. Part of the X-ray source is divided into two parts: a fan beam (upper portion) for transmission imaging and a narrow pencil beam (lower portion) for X-ray diffraction (XRD). Inset shows beam centre position defined based on diffraction rings cast on the HEXITEC detector. (**b**) Expanded view of the sample and detector showing the scattering geometry for an arbitrary pixel in the HEXITEC detector.

In this study the XRD data were collected using the pixellated diffraction (PixD) method^12^. The technique utilises a multi-element (pixellated) energy-dispersive detector. A histogram is recorded in each pixel based on the energies of the photons received. Provided that the scattering geometry (source, sample and detector configuration) is well-defined, each pixel can be assigned a fixed scattering angle (2*θ*) such that the energy (*E*) histograms can be converted into momentum transfer (*x*) histograms via Equation (2), where *hc* = 1.24 keV nm in convenient units, allowing the counts in all pixels to be summed together to give a single diffraction pattern. In this case *x* has units of nm^−1^. A schematic representation of the experimental setup is shown in Fig. 1a.

2 $$x\,=\,\frac{E}{hc}\,\sin \,\theta $$

Since X-rays are penetrating radiation they can be used to look at thick sections of tissue and hence could be used to enhance the conventional X-ray absorption imagery (e.g. mammography)^13–16^. Furthermore, compared to histopathological analysis, X-ray diffraction measurements are fast. PixD is a recent development in X-ray diffraction technology which allows diffraction signatures to be obtained with a small compact instrument in 10 s to 100 s of seconds^17^. Thus in-theatre analysis of resected tissue could be undertaken to look for involved (positive) margins.

Tissue which is mainly composed of fat has a sharp peak at ~1.1 nm^−1^. As the concentration of fibrous and connective tissue increases the intensity of the fat peak reduces and a new feature emerges at ~1.5 nm^−1^. The new feature is typically lower in intensity and broader than the fat peak, indicating that it is associated with less ordered tissues, in agreement with the findings of other authors^10,18^. The purpose of this study is not to investigate the shapes of individual structures within breast tissue. The aim is to look for correlations between the shapes of the XRD pattern measured from a bulk sample (which is assumed to contain a mixture of structure) with the histology of the same sample.

Alternative X-ray scattering based technologies currently being investigated for tissue classification include coded aperture coherent scatter imaging^19,20^ and diffraction enhanced CT^21–23^. This paper looks at the correlation between histopathological analysis and X-ray diffraction signatures for a range of breast tissue samples as a step towards establishing the viability of diffraction signature analysis for classifying tissue samples.

## Results

**Figure 2:**
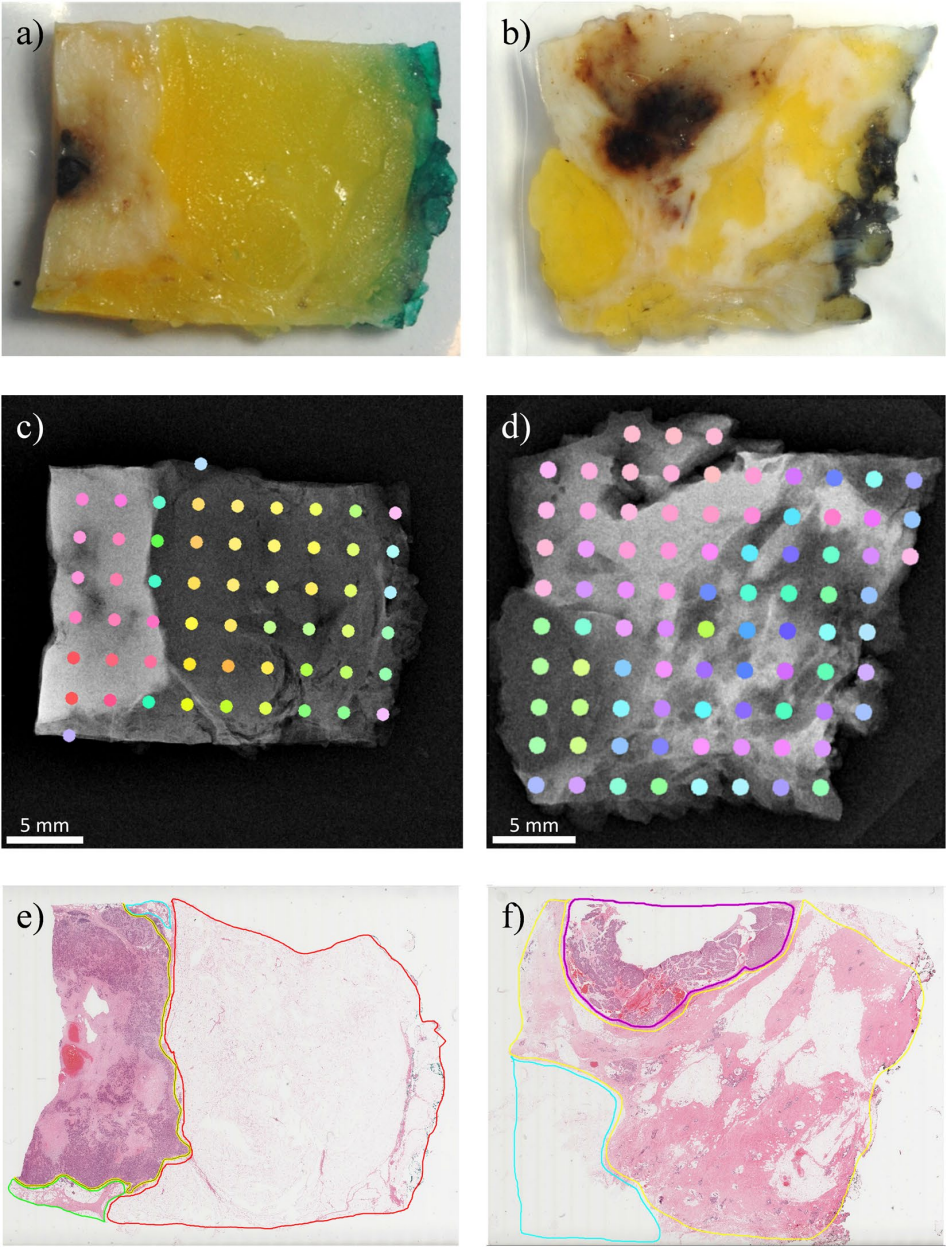
Comparison of two indicative samples showing (**a**,**b**) photographs of the samples as prepared, (**c**,**d**) colour-coded (based on principal component analysis (PCA) output) diffraction results overlaid on high resolution clinical mammography transmission X-ray images and (**e**,**f**) digital microscope images of a single slice through each sample. The boundaries demarcate the invasive tumour, normal breast tissue and fat as yellow, blue/green and red respectively in **e** and pink, yellow and blue respectvely in **f**.

A series of eight tissue samples were selected which were known to contain tumorous tissue, healthy/normal breast tissue and fat. Two tissue specimens are shown in Fig. 2a and b. The tissue was fixed in formalin which is known to have negligible effect on XRD measurements^10,20^. The dark green/grey edge is the ink applied to the surgical margin of the excised tissue prior to sectioning. The yellow tissue is fat and the white regions are dense tissue which includes normal/healthy breast tissue and/or carcinoma. Darker brown areas result from haemorrhage (due to preoperative diagnostic biopsy) or necrosis occurring at the centre of a tumour^24^. XRD measurements were taken at known positions across each sample (see Methods sections for further details). Principal component analysis (PCA) was applied to the diffraction measurements to classify the shapes of the scattering patterns. The samples then underwent histopathological analysis and the results were compared with the PCA classification of the XRD data.

X-ray measurements were made using the *miniPixD* instrument^17^ which has the capability to collect transmission X-ray images and XRD data of small samples. In this case the *miniPixD* images were simply used to define the sample position. The tissue samples were typically 30 × 25 × 5 mm in size. They were packaged in thin, sealed polythene bags, mounted on a sample holder and transmission X-ray images were captured. The images were used to define the tissue boundary and the positions to collect XRD measurements were automatically assigned (see Methods section for further details). XRD measurements were taken in transmission configuration (i.e. through the tissue and the containing bag). Measurements were also made of the polythene bag (without the sample) to assess the contribution of the bag to the overall scattering profile. Processing the PixD XRD data assumes that each pixel in the detector array has a single and well-defined scattering angle. This assumption holds true provided that the sample is relatively thin. As the sample becomes thicker, the lack of detector-side collimation means that the range of scattering angles each pixel can see becomes larger, having the effect of washing out the diffraction features. In this case the scattering angle and associated uncertainty has been calculated for the centre pixel as 5.83 ° [+0.42 °, −0.40 °], assuming the sample has a thickness of 5 mm (see Methods for further details).

PCA was performed on the full set of diffraction data from all samples combined. The spectrum of the polythene bag was subtracted from the tissue diffraction patterns as a background. However, it should be noted that removal of a constant value from all spectra had no effect on the PCA, which is only sensitive to differences. A window was applied in momentum transfer to restrict analysis to a region of interest between 0.7 and 3.0 nm^−1^. Features outside this range are related to the X-ray source properties (e.g. tungsten characteristic lines). It is also useful to impose a lower limit on momentum transfer to negate small variations in sample attenuation. Since it is low energy photons that contribute to low momentum transfer (at the scattering angles used here), small changes in sample thickness and composition can have significant effects on the magnitude of the signal in this region. This, in turn, can bias the PCA and smaller variations in spectral shape, due to real changes in the diffraction properties of the material, might be missed. Other than background subtraction, the spectra were not normalised or corrected for attenuation or spectral shape prior to PCA analysis since the factors would be the same across the samples measured.

**Figure 3:**
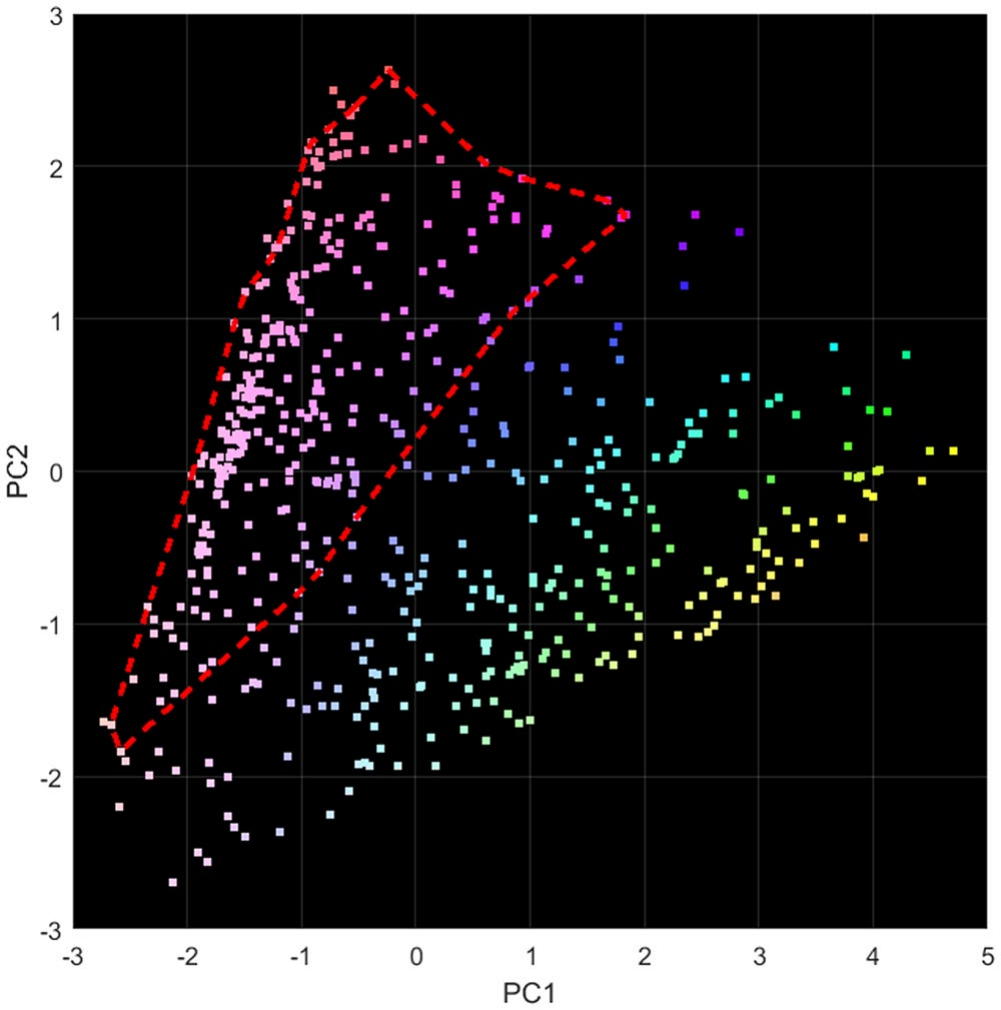
Principal component analysis (PCA) results based on the diffraction data (522 spectra) plotted with respect to PC1 and PC2. All positions within the samples that were identified as tumorous by histology are contained within the bounded region.

PCA showed that 99.0% of the variation between spectra could be described by the first two principal components (PC1 and PC2, 67.7% and 31.3% respectively). The spread of measurements with respect to PC1 and PC2 are shown in Fig. 3. It can be seen that the diffraction measurements of the breast tissue samples form a continuum with no distinct groups or clusters developing. This can be explained in terms of the samples being mixed specimens. XRD is an additive technique, meaning that the measured diffraction pattern of a mixed sample is a combination of the diffraction patterns of the individual components of the mixture. In this case a gradual transition from one material type to another would be expected in PC space for an ever changing composition of normal, fatty and cancerous tissue.

**Figure 4:**
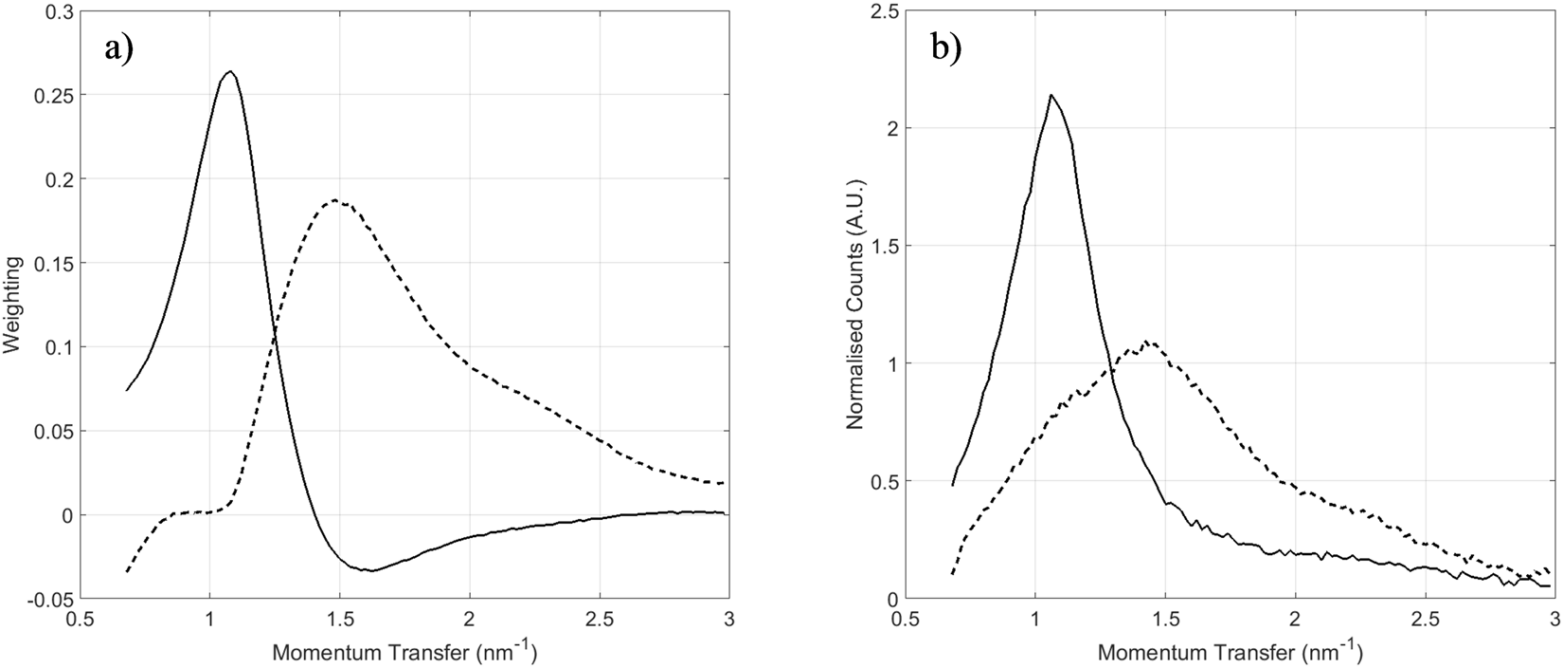
(**a**) Loading plots for PC1 (solid) and PC2 (dashed) and (**b**) X-ray diffraction (XRD) spectra having maximum PC1 score (solid) and maximum PC2 score (dashed).

The loading plots in Fig. 4a can be used to gain some insight into what the PCs are describing. Loadings describe the relationship between the original variable space and the PC space; linear combinations of these loading coefficients applied to unit vectors along each original axis results in a particular PC. They can therefore be used to identify diagnostic momentum transfer values which contribute to a particular PC. In this instance, the strong peak at 1.1 nm^−1^ for the PC1 loadings indicates that the spectra with more positive PC1 scores in Fig. 3 have this peak present. Similarly, the peak at 1.5 nm^−1^ in the PC2 loadings indicates that those spectra in Fig. 3 with more positive PC2 scores display this particular feature. This is demonstrated in Fig. 4b where the spectra with the maximum PC1 score and maximum PC2 score are plotted. As expected these exhibits strong features in the regions predicted by the loading plots.

In order to understand this distribution, the PCA results were compared to high resolution radiographs taken on a clinical mammography system (see Methods section for further details). For each sample the clinical mammography image and the *miniPixD* image were registered. Landmarks (distinctive features such as edges and corners) were identified in each image and a transformation matrix was calculated in Matlab. The transformation matrix was then used to map the XRD measurement positions onto the mammography images using a 2D projective geometric transform. To relate the XRD results to the histology of the samples, the points were colour coded based on their PC space coordinates. The coordinates were used to define the hue (H, Equation (3)) and the colour saturation (S, Equation (4)) on the HSV scale. A constant value (V = 1) was used for all measurements. It should be noted that the arguments contained in square brackets in Equations (3) and (4) were normalised to have values between 0 and 1.

3 $$H\,=\,1-0.9[PC1\times (1-PC2)]$$

4 $$S\,=\,0.25+0.75[PC1\times PC2]$$

The results of the colour coding is shown in Fig. 3 in PC space. The same colours were used to identify the PCA classification of the XRD with respect to the measured positions on the samples as shown in Fig. 2c,d.

After the XRD measurements were made, the samples were processed into paraffin wax, sectioned at 4 µm, mounted on glass slides and stained with haematoxylin and eosin. Digital micrographs were annotated and boundaries drawn around each tissue type present (e.g. carcinoma, normal breast tissue and fat). Examples are shown in Fig. 2e,f which can be compared to PCA classifications in Fig. 2c,d respectively.

## Discussion

**Figure 5:**
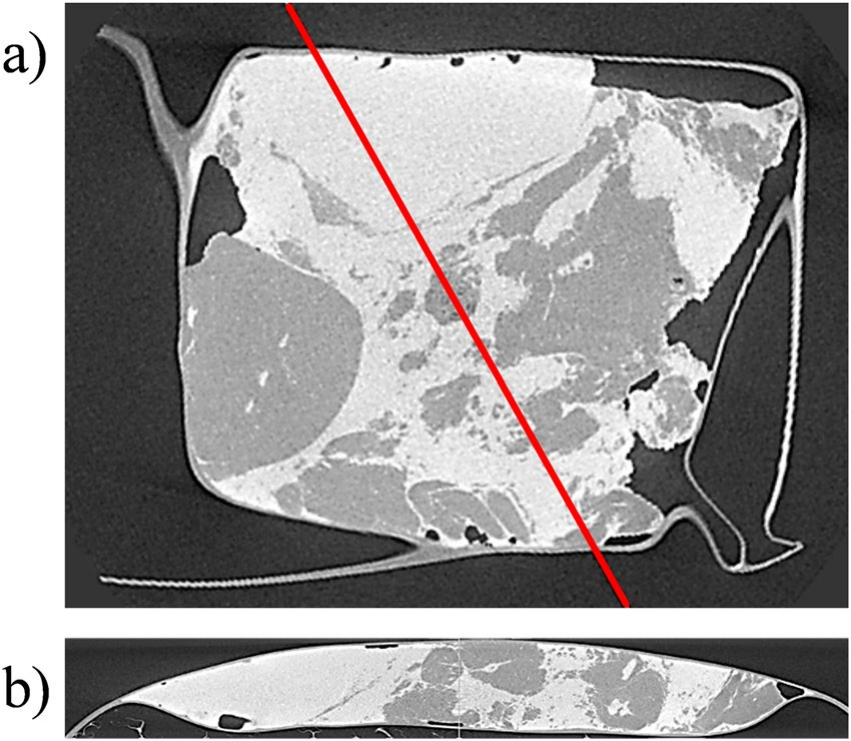
µCT images of a sample viewed (**a**) in the same orientation as shown in Fig. 2 (right hand column) and (**b**) as an orthogonal slice along the line shown in (**a**).

The ability to identify the presence of cancerous tissue in a bulk sample could be transformative for the diagnosis and treatment of breast and other cancers. The results presented here are encouraging since we have shown, through the application of PCA, that there is correlation between XRD and histological results. This is illustrated in Fig. 2. In some samples (e.g. Figure 2c) the transition from one tissue type to another is clear and is supported by the transmission X-ray image. In other samples (e.g. Figure 2d) the transition between tissue types is not so well defined and picking the tumour boundary in the transmission image is more difficult. It should be noted that the micrograph (Fig. 2e,f) corresponds to a thin layer (a few μm thick) through the sample, whereas the XRD measurements were made through the entire sample (a few mm thick), meaning that the histology shows only a fraction of what was actually interrogated by XRD. In order to demonstrate this, a μCT image of the sample shown in the right hand column of Fig. 2 is presented in Fig. 5. Figure 5a is a reconstructed slice at approximately the same depth as the pathology slide in Fig. 2f. High and low CT values are associated with higher density (normal tissues and tumour) and lower density (fat) tissues respectively. Figure 5b is an orthogonal slice through the sample along the line indicated in Fig. 4a. It can be seen that, even within a sample of a few mm in thickness, the tumour boundary shifts in depth such that the XRD would not see a ‘hard boundary’ as the pathology slide suggests. It can also be seen that the tissue type can vary significantly with the normal tissue being composed of dense areas (of stroma with or without glandular tissue) interspersed with pockets of fat. This supports the concept of tissue mixing being responsible for the spread of results observed in Fig. 3. It was also observed that the tumorous tissue (left side of Fig. 5b) is fat deficient compared to the surrounding normal tissue – a characteristic which has been reported by other authors^10^.

The pathology results for all eight tissue samples were compared to the XRD results. Any measured positions that fell within the boundaries indicated as invasive tumours by the pathologist were correctly identified by the colour-coded PC space plot (Fig. 3). It can be seen that these points are confined to the upper left portion of the plot within the indicated boundary. This gives confidence that XRD offers capability to indicate the presence of cancerous tissue in a bulk sample. While it has not been possible to differentiate tissue types (i.e. carcinoma, normal breast tissue and fat), the loading plots produced by the PCA analysis suggest that the variation in the data is associated with features consistent with these structures.

These results suggest that the shape and intensity of the X-ray scattering pattern can be used to discriminate between different breast tissue types which is supported by other X-ray scattering studies of breast tissue^7,8^. The correlation between the PCA analysis of the data and the histology of the samples is in general agreement with other published literature where similar comparisons have been made^20^. Further work is required to determine exactly what the important features are within the XRD spectra to make precise tissue type identification a possibility in the future.

## Methods

### Tissue samples

Tissue samples were obtained from the Tayside Tissue Bank (TTB), Dundee, UK. Permission and ethical approval to use the tissue was given by the Tayside Biorepository (TBR) Access Committee (application reference number TR000408). Authorisation for TBR to grant ethical approval is delegated from the Local Research Ethics Committee. All work with the tissue was carried out in compliance with all applicable laws, regulations, guidelines and approvals as outlined in the TTB terms and conditions.

Eight tissue samples (from four individuals) were acquired. All patients had undergone therapeutic breast surgery for treatment of cancers larger than 20 mm in diameter, and all had given informed consent for use of their tissues for research purposes. Resected specimens were formalin-fixed according to standard protocol. After formalin fixation, samples were selected such that the blocks taken for research purposes included macroscopic tumour, the peritumoural boundary and more distant macroscopically normal breast tissue. The blocks measured approximately 30 × 25 mm and were up to 5 mm thick. All blocks were sealed in a polythene bag from which most air had been removed for easy handling and to preserve their condition.

### Image guided X-ray diffraction

Image guided XRD measurements were made using the *miniPixD* instrument^17^. The instrument consists of a compact X-ray tube, a sample position system, a transmission X-ray detector and a pixellated energy dispersive X-ray detector. The experimental setup is shown schematically in Fig. 1. The X-ray tube settings were maintained at 72 kVp and 1.0 mA throughout the measurements. Beam filtration was provided by the 120 μm beryllium exit window only. A 2 mm thick lead collimator was place 37 mm from the X-ray tube focal spot and was designed to define a pencil beam (through a 0.6 mm pinhole) and a fan beam (through a 1 mm slot). The pencil beam was further defined by a second 0.6 mm pinhole collimator placed 235 mm downstream. The fan beam was incident on the linear strip detector (0.2 mm photodiode pixels with Gadox scintillator).

The PixD method^12^ was used to make the XRD measurements. The technique utilises a cadmium telluride (CdTe) sensor bump bonded to an advanced application-specific integrated circuit (ASIC) in a square configuration, offering 80 × 80 pixels with a 250 μm pitch^25^. The detector was biased to −475 V. The bias voltage was cycled every 60 s to avoid polarisation of the CdTe sensor. Data were collected with 0.25 keV energy bins in the range of 0 to 100 keV. A low energy threshold of 5 keV was used to eliminate detector noise. The pixel registers were read out at a rate of 1.5 kHz and the data were processed on a frame-by-frame basis. If an X-ray interacts with the CdTe sensor near a pixel boundary it is possible that the liberated charge is spread across and recorded by adjacent pixels. This effect is called charge sharing and the result is that a single photon event is wrongly recorded as two separate, lower energy events^26^. A correction was applied whereby events demonstrating charge sharing were rejected from the analysis.

The XRD data were collected in transmission mode (i.e. through the tissue) for 300 s at each location. The incident pencil beam size at the sample was approximately 1 mm diameter such that the volume interrogated per measurement was approximately 4 mm^3^ (cross-sectional area of the beam multiplied by the sample thickness) in this case. The distance between the second beam shaping collimator and the sample position was measured to be 122 mm and the beam divergence was calculated geometrically to be 0.2 °. The pixellated sensor was position such that the primary pencil beam just passed one corner of the detector which measured scatter for angles between 0.1 ° and 11.2 °. A generalised schematic of the scattering geometry is given in Fig. 1b. A conical X-ray beam is incident on a sample of thickness 2 *T*. The beam has a divergence angle 2*φ* and a diameter 2*r* at the centre of the sample. An arbitrary pixel is located distance *D* from the centre of the sample and distance *d* from the beam central axis. In the data processing the nominal scattering angle (2*θ*_*nom*_) of each pixel is used in the conversion to momentum transfer space via Equation 2. In reality a pixel records photons from a range of scattering angles between *2θ*_*min*_ and *2θ*_*max*_. Equations (5–7) are derived from the geometry in Fig. 1b and can be used to calculate the scattering angle uncertainty for an arbitrary pixel.

5 $$\tan \,2{\theta }_{nom}\,=\,\frac{d}{D}$$

6 $$\tan (2{\theta }_{min}+\phi )\,=\,\frac{d-r+T\,\tan \,\phi }{D+T}$$

7 $$\tan (2{\theta }_{max}-\phi )\,=\,\frac{d+r+T\,\tan \,\phi }{D-T}$$

By way of example, consider the current experimental setup and the pixel located at the centre of the detector such that *d* = 14.1 mm, *D* = 138 mm, *2* *T* = 5 mm, *2r* = 1 mm and *2φ* = 0.2 °. The scattering angles derived from Equations (5–7) are, *2θ*_*nom*_ = 5.83 °, *2θ*_*min*_ = 5.43 ° and *2θ*_*max*_ = 6.25 °, thus giving this pixel a scattering angle of 5.83 ° [+0.42 °, −0.40 °]. The uncertainty in scattering angle, and in the detector energy resolution, determines the uncertainty in momentum transfer. The energy resolution of the detector used here has been measured previously to be 1.6 keV at 60 keV (FWHM)^17^. By Equation 2, the nominal momentum transfer value for a 60 keV photon received at the centre pixel (as described above) would be 2.46 nm^−1^. The bounds of the momentum transfer value can also be calculated using the upper and lower energy and scattering angle values. The worst case upper and lower momentum transfer values are 2.26 nm^−1^ and 2.67 nm^−1^ respectively.

The system was calibrated to determine the sample to detector distance and the position of the beam centre with respect to the pixels. Accurate knowledge of these parameters is essential for obtaining accurate results. A 3 mm thick sample of caffeine was used for calibration. The caffeine was placed at the same location on the sample stage as the tissue specimens and diffraction data were collected. An image was formed by taking a narrow energy slice from the detector data which shows one of the diffraction rings. The beam centre was determined by fitting a circle through the diffraction ring (see Fig. 1a). The diffraction pattern of caffeine has two prominent peaks at 0.67 and 1.47 nm^−1^. The sample to detector distance was adjusted iteratively to match the peaks to the known positions. The optimal beam centre position and sample to detector distance were used to analyse the tissue sample data.

### Clinical mammography imaging

High resolution transmission X-ray images were acquired using a Selenia Dimensions (Hologic, Inc.) clinical mammography unit set at 26 kV, a target and filter combination of tungsten and rhodium respectively and a magnification of 1.8. The mA and exposure time were selected by the system, operating under the automatic exposure control (AEC) mode of ‘auto-time’. The 4^th^ AEC area was selected and the samples positioned accordingly. The Modulation Transfer Function was determined by measuring the Edge Response Function. The average resolution was found to be 10.62 lines mm^−1^.

### μCT imaging

Computed tomography data were acquired using a Nikon XT H 225 micro-CT system (Nikon Metrology). The system was operated in reflection configuration with Mo target (40 kVp and 400  μA, 16 W) and the X-ray beam was filtered by the way of a 0.25 mm Mo foil. To obtain a good signal-to-noise ratio, a total of 3176 radiographs were acquired over a 360° sample rotation range with an exposure time of 1 s per projection. The sample was placed between the X-ray source and an amorphous silicon detector (PerkinElmer 1620 detector, 2000 × 2000 pixels at 200 μm pitch) providing a voxel resolution of ~23.9 μm at a geometric magnification of 8.37.

### Histopathology analysis

Histopathology was conducted on the samples after the XRD measurements were taken. Following the acquisition of the XRD data, the samples were processed into paraffin wax using standard techniques, sectioned on a microtome at 4 µm, mounted on a glass microscope slide and stained with haematoxylin and eosin (the standard stains for diagnostic microscopy). An Aperio ScanScope XT microscope (Leica Biosystems) was used to capture high resolution images.

### Data availability

The datasets generated during and/or analysed during the current study are available from the corresponding author on reasonable request.
